# Study of working from home: the impact of ICT anxiety and smartphone addiction on lecturers at NIPA School of Administration on job performance^☆^

**DOI:** 10.1016/j.heliyon.2022.e11980

**Published:** 2022-12-06

**Authors:** Adi Suryanto, Rachma Fitriati, Sela Inike Natalia, Andina Oktariani, M. Munawaroh, Nurliah Nurdin, Young-hoon AHN

**Affiliations:** a National Institute of Public Administration, Indonesia; b Universitas Indonesia, Faculty of Administrative Science, Indonesia; c IPB University, School of Business, Indonesia; d Jakarta State University, Faculty of Economy, Indonesia; e The Presidential Committee on Autonomy and Decentralization, Republic of Korea

**Keywords:** ICT anxiety, Smartphone addiction, Job performance, COVID-19, Work from home, PLS-SEM

## Abstract

The COVID-19 pandemic has significantly impacted on the working system, shifting working from office (WFO) into working from home (WFH) practice that requires employees to be skillful in using technology to support their work activities. However, this condition can affect job performance. This study aims to analyze the impact of ICT anxiety and smartphone addiction on job performance of all lecturers at NIPA School of Administration (Jakarta, Bandung, and Makassar). This study applied a quantitative method with a total sampling technique and conducted a survey on 135 respondents using an online questionnaire. Furthermore, this study employed job demands and resources theory as well as PLS-SEM to analyze five variables (ICT anxiety, smartphone addiction, interruption, job efficacy, and job performance) and to test seven hypotheses. The findings show that there is a positive relationship between ICT anxiety and interruption while interruption has negative influences on job efficacy and job performance. Therefore, this study recommends the facilitation of knowledge sharing related to ICT competence or literacy. In addition, NIPA should improve the security guarantees of the intellectual rights of the lecturers in relation to the choice of technology and integrate the demands of ICT needs with administrative-technical procedures.

## 1 Introduction

Job Demands and Resources (JD-R) theory examines the former that can trigger stress and the latter that can increase work motivation (Prodanova and Kocarev, 2021). In this regard, the JD-R theory is related to the use of technology that is currently a significant component in work activities, considering that technology can be a demand or even a resource that influences employees.

The COVID-19 pandemic has significantly impacted on the working system, shifting working from office (WFO) into working from home (WFH) practice which poses a major challenge for employees. According to Cabinet Secretariat of the ​Republic of Indonesia in 2020, the WFH system has been implemented in Indonesia since March 2020.

Furthermore, the Minister of Administrative and Bureaucratic Reform has issued Circular Letter Number 19 of 2020 on Adjustment of State Civil Apparatus (Civil Service) Working System to Prevent the Surge of COVID-19 in Government Agencies. Conforming to this policy, all civil servants have to carry out their official duties from home. The WFH policy in Indonesia is dynamic in coherence with the restrictions on community activities in response to the COVID-19 surge.

Shen et al. (2020) reveal that the COVID-19 pandemic hinders organizational goals and negatively affects organizational performance. The COVID-19 pandemic triggers the emergence of fear, anxiety, and stress that are part of interruption, leading to the decrease in task completion and eventually low employee performance (Li and Lin, 2019; Vo-Thanh et al., 2020). Stress can also cause low employee contribution and productivity, resulting in low organizational performance (De Clercq et al., 2017).

Another factor closely related to job performance is efficacy, particularly in task completion that involves a large-scale working environment (Liang et al., 2020). In this regard, task completion time, implementation schedule, and task completion actions can explain the success level of employee job performance (Andreassen, 2015; Taylor et al., 2013).

Employees who work from home face a great challenge that requires self-preparation and technology (Rachmawati et al., 2021). The use of technology aims to facilitate work activities and increase productivity even though the work activities are conducted from home. Belzunegui-Eraso and Erro-Garcés (2020) reinforce that technology supports the implementation of a flexible working system with faster working time, efficiency, and mobile working activities.

However, other studies have shown that use of technology can negatively affect employee productivity (Mazidi et al., 2020). Turel et al. (2011) state that constant use of technology can lead to high workload, addiction to use of technology, and particular problems for employees such as fear or discomfort. It strengthens the hypothesis that use of technology at work has negative impacts, for instance, fear, discomfort, and addiction.

Fear or discomfort experienced by employees regarding technology is referred to as ICT anxiety (Barbeite and Weiss, 2004). ICT anxiety can arise when job demands require employees to optimally use technological tools to improve work results and efficiency (Alahakoon and Somaratne, 2018; Chhabra et al., 2020; Van Steenbergen et al., 2018). ICT anxiety can be an obstacle in the implementation of the WFH system since WFH activities are only possible with technological assistance (Khan et al., 2021; Mac Callum et al., 2014).

Besides ICT anxiety, another negative impact is an addiction to smartphone use. Smartphone addiction can be defined as a form of individual dependency due to excessive use of smartphones (Richard et al., 2020) that can negatively affect individual behavior, social relationships, and time management (Hsieh et al., 2020). Continuous use of smartphones can cause addiction (Brem et al., 2021; Li and Lin, 2019; Turel et al., 2011) which influences the ability of employees to carry out and control the implementation of their work tasks, affecting the achievement of company performance and goals. Smartphone addiction also affects the level of concentration and collaboration of the employees (Li and Lin, 2019).

This study aims to analyze the impact of ICT anxiety and smartphone addiction on job performance of all lecturers at NIPA School of Administration with intervening variables, namely interruption and work efficacy. NIPA School of Administration is selected as the unit of analysis because this Polytechnic is a government-affiliated college outside the Ministry of Education, Culture, Research, and Technology of the Republic of Indonesia. NIPA School of Administration was firstly established as the State Administrative Sciences College, whose students were Civil Servants with associate and bachelor degree. To meet the needs of employees with associate degree of administration, the Academy of Administrative Sciences was established in 1964 in Jakarta and accepted civil servants with high school degree or equivalent. The State Administrative Sciences College and the Academy of Administrative Sciences were then integrated into the Graduate School of NIPA School of Administration as specified by Presidential Decree Number 5 of 1971. NIPA School of Administration was then designed to organize academic and professional education programs in administrative science for government employees in compliance with Presidential Decree Number 10 of 1999. Nowadays, three NIPA School of Administration in Indonesia are located in Jakarta, Bandung, and Makassar.

The impact of ICT anxiety and smartphone addiction becomes a challenge for the learning system at NIPA School of Administration, whose entire academic community consists of government employees (such as State Civil Apparatus and employees of State and Local-Owned Enterprises), the Indonesian National Police, and the Indonesian National Armed Forces. The WFH policy forces lecturers and students to implement the studying from home system by utilizing ICT. It is a challenge because students of NIPA School of Administration are not familiar with the learning system using technology, such as video conferencing and online teaching materials.

This study provides an update of performance studies during the COVID-19 pandemic that has had a major impact on the implementation of distance learning systems. In fact, the selection of ICT anxiety and smartphone addiction as variables makes this study interesting. These two variables are unique, as they seem to have opposite meanings but can greatly affect the performance of the lecturers. Discussions about job performance amid the COVID-19 pandemic have also become renewable considering the increasing human activities and interactions with technology.

## 2 Literature review and theory

### 2.1 Conceptual review

The current use of technology influences work arrangements, which is referred to as the New Ways of Working or NWW (Van Steenbergen et al., 2018). The work system becomes more flexible and can be done anywhere, reducing workload and employee stress. Job demands can indeed decrease when a company uses technology in most activities. However, it should be underlined that employees may need time to adjust their work needs to technology (Van Steenbergen et al., 2018). In addition to its impact on job demands, the use of new ways of working using technology also affects job resources, by enabling more effective communication that strengthens the working relationship between superiors and subordinates. However, it apparently also results in a decline in career development since work flexibility renders employees lacking opportunities to learn and control overwork (Van Steenbergen et al., 2018).

The use of technology is also associated with job demands and job resources, as discussed by Prodanova and Kocarev (2021). Anxiety (job demands) and addiction (job resources) in the use of technology have an impact on employee work performance; therefore, technological tools (both hardware and software) must be used according to the provisions and capacities to control and reduce their negative impact on work performance. Job efficacy also influences job performance, but companies need to pay attention to interruption that can decrease job performance.

When discussing about technology tools that can support work activities, we can mention smartphones. Smartphones now allow more effective working without being limited by space and time, hence the chance for an individual who uses a smartphone at work to experience addiction. Li and Lin (2018) explain that dependence on smartphone is related to job performance because it can increase work efficiency and employee workplace social capital. Employee workplace social capital increases when employees interact with each other about work via smartphones. It will cause a social response to build a more significant social interaction (Li and Lin, 2018). However, it cannot increase the workplace social capital of the company because it is not considered an outcome of communication dependency.

Based on several aforementioned studies, it is evident that technology and job performance are interrelated. Employees who use technology tools effectively can increase work collaboration, self-confidence, and work knowledge which are the basis of work cooperation that can mediate work performance (Pitafi et al., 2018).

The use of technology is increasingly perceptible during the COVID-19 pandemic, where most work relationships are virtual, resulting in the need for companies to observe how employees build quality work that in return will affect job performance (Narayanamurthy and Tortorella, 2021). Narayanamurthy and Tortorella (2021) explain that, based on the Social Construction of Technology (SCOT) theory, the work environment when working from home can improve the quality of work and employee performance. In addition, implications of work during the pandemic directly influence employee work performance because work activities are carried out remotely. The use of technology during WFH cannot mediate the work environment of the employees to quality work and cannot mediate job insecurity to the quality of work results. However, the use of technology in work activities with the WFH system can mediate virtual connectedness to the quality of work results (Narayanamurthy and Tortorella, 2021).

### 2.2 Hypothesis development

#### 2.2.1 Job demands and resources theory (JD-R)

The concept of job performance is related to job demands and resources. Referring to the opinion expressed by Bakker and Demerouti (2014), job demands and resources theory (JD-R) is a concept of development from job design and job stresses theory. The JD-R theory covers two aspects: job demands that can trigger employee stress and job resources owned by a company that can increase work motivation of employees. Job demands refer to the existing physical, psychological, and social aspects of an organization related to company costs (Demerouti et al., 2001).

Meanwhile, job resources refer to the physical, psychological, and social aspects owned by an organization that can functionally motivate employees to achieve work goals, reduce job demands and costs incurred, and encourage employee desire to develop themselves (Bakker, 2011; Bakker and Demerouti, 2007). This theory can also explain the environment and the implementation of work activities that can affect the results or achievements of the company.

Bakker and Demerouti (2014) explain several aspects of the JD-R theory, including flexibility and two processes that are related to achieving employee welfare and influencing employee performance. Employees with high job resources can minimize the effects of perceived job demands. Another aspect of the JD-R theory is personal resources, namely employee self-evaluation, which is related to the ability of the employees to succeed in work activities as it is related to motivation, performance, self-efficacy, and job satisfaction (Hobfoll et al., 2003). Another aspect is personal demands, namely the determination of the employees to carry out work activities and improve their performance, such as the level of perfectionism and expectations of performance standards (Bakker and Demerouti, 2017; Barbier et al., 2013). Based on Bakker and Demerouti (2007), the JD-R theory is used to project employee welfare; influence company commitment, job satisfaction, connectedness, and work engagement; and predict several consequences of work decisions taken by employees.

#### 2.2.2 ICT anxiety

ICT anxiety can be defined as barriers or personal problems when using technology (Maican et al., 2019; Mitzner et al., 2010). Hsieh et al. (2020) define ICT anxiety as a feeling of discomfort when using technology and a reluctance to use new technology. Meanwhile, Saadé and Otrakji (2007) explain that the impact of using technology in the past and present and the decisions to use technology in the future will influence the anxiety of an individual in using technology.

North and Noyes (2002) provide another term for ICT anxiety, namely technophobia, defined as anxiety when interacting with technology either now or in the future. North and Noyes (2002) further explain that technophobia is an overall negative attitudes towards computer operations, both fear of social impacts and self-criticism that will result from using technological devices and fear of using technology in the future. According to Saadé and Kira (2009), there are three types of anxiety in which ICT anxiety is included in concept-specific anxiety because it is related to specific situations, namely when interacting or using technology. Saadé and Kira (2009) also explain that ICT anxiety is related to the belief of an individual in using technology.

Czaja et al. (2006) explain that an individual with anxiety over using technology tends to use technology rarely, or never. Ellis and Allaire (1999) also argue that an individual with ICT anxiety does not desire to use technology. These two opinions are relevant. After all, ICT anxiety influences individual decisions to use technology (Meuter et al., 2003) because it significantly influences individual attitudes (Celik and Yesilyurt, 2013). In addition, anxiety over using technology can prevent the individual from using other technologies (Balta-Ozkan et al., 2013; Venkatesh et al., 2012).

Saadé and Kira (2009) also explain that ICT anxiety will affect individual productivity, welfare, and social relationships. An individual with a high level of ICT anxiety can cause problems in daily work productivity due to ineffective and inefficient performance (Bai, 2019). ICT anxiety also affects work processes that will affect the performance results of the company (Belzunegui-Eraso and Erro-Garcés, 2020), especially when there is an increase in the use of technology during the COVID-19 pandemic (Elhai et al., 2020). When using technology at work, self-confidence will influence self-capacity to achieve efficient performance. This later can optimize the use of new technology to efficiently complete the given work and continuously integrate new technology in completing the work (Mac Callum et al., 2014).

H.1 There is a positive relationship between ICT anxiety and interruption.

H.2 There is a negative relationship between ICT anxiety and job efficacy.

#### 2.2.3 Smartphone addiction

Smartphone addiction is an addictive behavior with a negative connotation (Li and Lin, 2019). Kardefelt-Winther et al. (2017) describe the concept of smartphone addiction as the failure of an individual to control their behavior that causes addiction. Ting and Chen (2020) explain that smartphone addiction is a compulsive behavior that causes excessive interaction between humans and smartphones. Smartphone addiction will be different from one individual to another due to differences in smartphone usage. Several researchers refer smartphone addiction as excessive interaction between humans and technology, causing problems in psychological aspects in relation to behavioral changes, such as withdrawing from social activities and losing self-control when using smartphones (Park et al., 2013; Bian and Leung, 2015; Li and Lin, 2018).

Referring to the media system dependency (MSD) theory (Ball-Rokeach and DeFleur, 1976), smartphone addiction is the dependence of an individual on their smartphone to fulfill a goal. Obviously, the goal will differ between one individual and another (Li and Lin, 2016). The Mass Communication theory published in Li and Lin (2019) explains the two levels of dependence on smartphone use, namely the macro and micro levels. Macro-level dependence is related to various media as well as economic, political, and social systems connected via smartphones. Meanwhile, micro-level or individual-level dependence is related to the interaction between individuals.

Ting and Chen (2020) identify three factors causing smartphone addiction in an individual: environmental, psychological, and social factors. Derks and Bakker (2014) state that individuals using smartphones have a higher risk of addiction. Smartphone addiction can cause negative impacts physically, psychologically, and socially (Ting and Chen, 2020). The initial purpose of using smartphones is inseparable from the need of an organization or company to effectively and efficiently achieve its performance. However, the high level of smartphone use in daily activities can lead to the bad effects of smartphone use (i.e. addiction) which will later affect the performance and work results of the individual.

H.3 There is a positive relationship between smartphone addiction and interruption.

H.4 There is a negative relationship between smartphone addiction and job efficacy.

#### 2.2.4 Interruption

Interruption can be interpreted as demands that interfere with work and affect the emotional aspects of employees and the level of fatigue when working (Mazmanian et al., 2013). Another opinion explains that interruption can disrupt the flow and process of working on tasks, causing employees to make wrong decisions (Dieckmann et al., 2007). Jett and George (2003) explain that interruption is a condition that hinders or delays the achievement of a work goal. In addition, interruption arises from within an individual and can also be influenced by activities around them.

Interruption negatively influences employee welfare as it causes emotional problems that hinder work activities and the achievement of work goals of the company. Therefore, employees have to spend extra effort to adjust after experiencing interruption, including longer time to optimally complete each task. In addition, employees tend to be more focused on understanding and completing new work tasks, despite their attempt to adapt the tasks previously affected by distractions. Interruption results in the inability of employees to complete the work tasks on time due to procrastination on the given work tasks, rendering task completion ineffective and inefficient. Another impact of interruption is failure when working (Elfering et al., 2014), such as declining work involvement, lacking work control, reducing work focus, and disrupting work routines (Wang and Suh, 2018). The most significant impact of interruption in work is stress, frustration, and other types of negative emotions that affect employees. Interruption from higher education in Indonesia comes from changing into online teaching system and delaying or even eliminating some academic activities (Lestari et al., 2020).

H.5 There is a negative relationship between interruption and job efficacy

H.6 There is a negative relationship between interruption and job performance.

#### 2.2.5 Job efficacy

Job efficacy is the belief that employees can perform any given tasks (Bandura & National Institute of Mental Health, 1986). According to Bandura (1997), job efficacy is related to employee well-being, decreased stress levels, positive emotions, and better self-adaptation. Job efficacy is explicitly grouped based on individual behavior that aims to control the work environment and activities (Schaubroeck et al., 2000). Several researchers also have the same opinion regarding the definition of job efficacy, namely the assessment of a cognitive ability of an individual to perform a better job (Bozeman et al., 2001; Lubbers et al., 2005). The role of an individual with high job efficacy in work activities will undoubtedly be larger compared to employees with lower job efficacy (Lubbers et al., 2005). Lubbers et al. (2005) explain that job efficacy will influence job performance because employees with high job efficacy will optimize task completion and their abilities.

Fida et al. (2015) explain that job efficacy is the belief in the ability of an employee to deal with situations in an uncertain work environment. Based on the job demands-resources theory, job efficacy is included in the type of company resources that can help reduce the negative impact of job demands (Bakker and Demerouti, 2007). An individual with a high level of efficacy also has high self-confidence to overcome problems and changes in work situations (Zhang et al., 2021). According to Bandura (2001), job efficacy can increase individual control over work activities. In addition, several opinions state that job efficacy can trigger the formation of critical strategies by employees, better time management, and well utilization of both existing and new technologies (Miraglia et al., 2017; Schenkel et al., 2019; Zainab et al., 2017).

The impact of high efficacy in an employee is an increase in job performance, as job efficacy is a benchmark for assessing the ability of employees to manage and complete every work activity, aiming to achieve optimal company performance (Mosley et al., 2008). Lent et al. (1994) even state that job efficacy is an essential aspect of organizational commitment to improving company performance. Carter et al. (2018) explain that job efficacy will affect the competence and skill improvement and motivate employees to use their experience as an impetus to optimize performance. An increase in abilities surely will lead to the success of the company.

H.7 There is a positive relationship between job efficacy and job performance.

#### 2.2.6 Job performance

In simple terms, job performance can be defined as the work of the employees to produce good results or not (Winter, 1980), a nearly similar definition as productivity and work efficiency, profits, and the achievement of company goals (Downs and Moscinski, 1979). Job performance is one part of employee work behavior related to organizational goals, which is an achievement (Omotunde and Alegbeleye, 2021). In this regard, the achievement refers to the implementation of tasks according to the agreed policies and the ability of employees. The achievement of organizational goals is highly dependent on employee performance.

Murphy introduces four factors affecting job performance, namely task behaviors, interpersonal behaviors (communication and cooperation between employees), downtime behaviors (behaviors that avoid work), and destructive/hazardous behaviors (behaviors that can reduce work productivity) (Dillon and Pellegrino, 1989).

Furthermore, Campbell introduces eight factors that affect job performance, namely (1) job-specific task proficiency, (2) non-job-specific task proficiency, (3) written and oral communications, (4) demonstrating effort, (5) maintaining personal discipline, (6) facilitating peer and team performance, (7) supervision, and (8) management and administration. These eight factors are popularized by having different patterns with a relatively high degree of variation because they adapt to the type of work (Koopmans et al., 2011). The learning and exploration process in an organization have an effect in maintaining and improving organizational performance (Suryaatmaja et al., 2020).

Borman & Motowidlo (1993) also explain two dimensions of job performance in a company: task performance and contextual performance. Task performance is related to the technical aspects of the organization in supporting the achievement of company goals through several processes of production, both goods and services. Task performance is related to the work tasks of employees. In comparison, contextual performance is a pattern of employee behavior in work activities leading to psychological and social aspects. Although it does not lead to the main task of employees, contextual performance is significant as it can shape the social and psychological aspects of the company to help optimize the critical thinking processes of employees. This study examines the effect of several variables such as ICT anxiety, smartphone addiction, interruption, and job efficacy on job performance (Table 1).

**Table 1:** Hypothesis.

No	Hypothesis
H.1	There is a positive relationship between ICT anxiety and interruption
H.2	There is a negative relationship between ICT anxiety and job efficacy
H.3	There is a positive relationship between smartphone addiction and interruption.
H.4	There is a negative relationship between smartphone addiction and job efficacy
H.5	There is a negative relationship between interruption and job efficacy
H.6	There is a negative relationship between interruption and job performance
H.7	There is a positive relationship between job efficacy and job performance

## 3 Materials and methods

### 3.1 Research design

**Figure 1:**
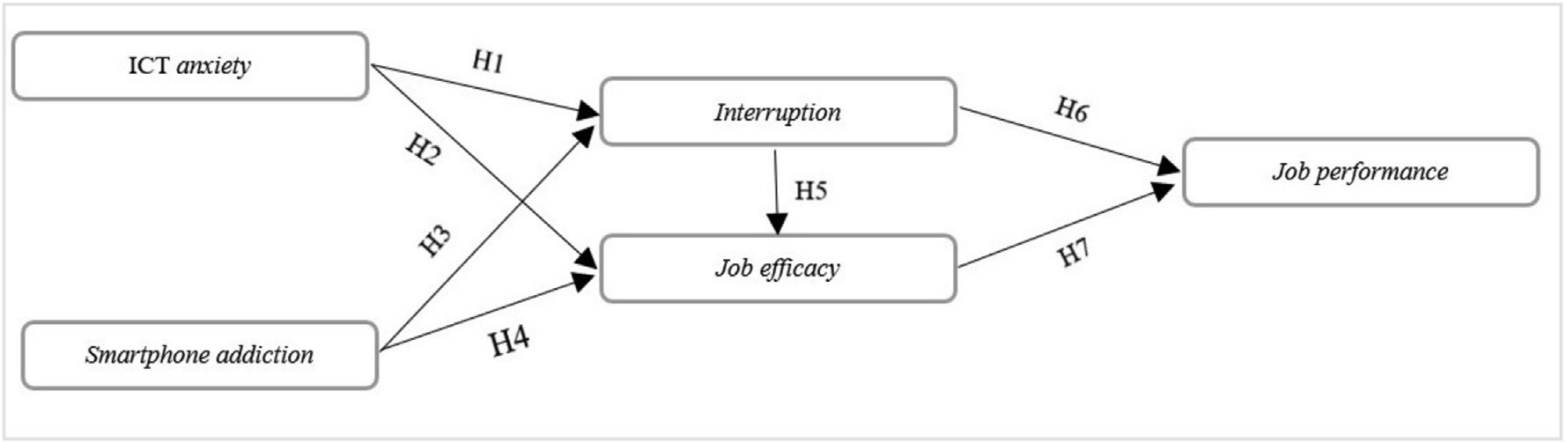
Research model.

This study applied a quantitative approach, namely a deductive study, as it applied theory as the primary basis and later combined it with the results of processed data (Creswell and Creswell, 2018; Neuman, 2014). The quantitative approach combines and harmonizes deductive logic with an empirical study to find and explain the behavior and patterns (Neuman, 2014). This study employed theories related to the variables, namely the theory of job demands-resources, ICT anxiety, smartphone addiction, interruption, job efficacy, and job performance, which also corresponding to the primary reference journals, as depicted in Figure 1.

This study applied a case study survey research as it discussed social phenomena that occur in the current period (the pandemic period), used the entire population, and distributed the questionnaire online (Yin, 2018). This study employed a cross-sectional method on a total population size of 135 people, namely all permanent lecturers at NIPA School of Administration in Indonesia.

Non-parametric statistics were employed on Likert-scale ordinal data obtained through categorization or classification, implying that the data are not normally distributed and thus eliminating the need for normality assumption and outlier testing (Kraska-MIller et al., 2013). The data were analyzed using Partial Least Square (PLS) method which does not assume a specific distribution for parameter estimation. Thus, tests for the significance of parameter are unnecessary as well (Albers, 2010).

This study also applied partial least squares structural equation modeling (PLS-SEM) to analyze data and provide evidence of reliability and validity. The application of PLS-SEM is highly recommended when the data have a limited number of samples while the model built is complex (Purwanto et al., 2021). PLS-SEM algorithms are generally claimed to perform particularly well with small samples and non-normal data (Hair et al., 2011). PLS-SEM works efficiently with small sample sizes and complex models and provides practically no assumptions about the underlying data (Cassel et al., 1999).

PLS-SEM can easily handle reflective and formative measurement models, as well as single-item constructions, without identification problems. In addition, PLS-SEM generally achieves a high level of statistical power with a small sample size. Therefore it can be applied in various research situations (Hair et al., 2017b). The advantage of PLS-SEM is that research can use very specific populations (Sander and Lee, 2014). PLS-SEM is a good alternative because it is more robust, meaning that the model parameters do not change much when a new sample is taken from the total population (Geladi and Kowalski, 1986).

The questionnaire consists of screening questions, the identities of respondents, and questions related to variables. The questionnaire used a five-point Likert-type scale as an appropriate assessment tool for studies using an interval scale to ensure that the same interval will apply among response categories (Creswell, 2015). The Likert rating scale is applied to measure psychometric indicators using a fixed-format rating scale (Robinson, 2018), which is the focus of this article. Scale is one of the most widely used instruments to measure opinions, preferences, and attitudes, which is also in accordance with this research (Likert, 1932). The Likert scale used is ordinal data that determine the rating of a perception indicator (Lee et al., 2010). This ranking considers the order of objects from the largest to the smallest or from the highest to the lowest. The Likert rating scale can be ranked form two to eleven response points, in which reliability, criterion validity, and the ability to discriminate between ratings of participants increase as the number of response points increases (Preston and Colman, 2000).

This study applied a five-point Likert-type scale based on the type scale that is a prerequisite of psychometric scales (Kline, 2000). A five-point Likert-type scale was selected because this scale can fulfill indicator reliability and allow the respondents to easily understand and distinguish each point on the scale, allowing easier information processing of the obtained data (Preston and Colman, 2000). The scale can be required to capture the richness of multidimensional variables. In addition, the determination of the scale is also based on the variables investigated, questionnaire space limits, or participant characteristics. The maximum number of items per scale will depend on the complexity of the variable being measured. A five-point Likert-type scale was selected by investigating cultural as well as other demographic differences such as gender and age in this study (Leung, 2011). Furthermore, a five-point Likert-type scale are preferable to the general public (Weijters et al., 2010) and able to express the statements of the respondents precisely and comfortably (Krosnick and Fabrigar, 1997). The Midpoint scale has shown good validity against equivalent full-scale versions (Nagy, 2002). Moreover, some researchers suggest that single-item measures may be preferred to multi-item scales (Postmes et al., 2013).

### 3.2 Participants

This study used a total sampling technique (Pitner et al., 2020), meaning all of the population that comprises a total of 135 lecturers of NIPA School of Administration in Jakarta, Bandung, and Makassar. No missing data were found since the respondents could answer all research questions.

All respondents have implemented online teaching method in response to the issuance of a public policy that enforces the WFH system in dealing with the pandemic, namely Circular Letter Number 19 of 2020 on Adjustment of State Civil Apparatus (Civil Service) Working System to Prevent the Surge of COVID-19 in Government Agencies by the Minister of Administrative and Bureaucratic Reform.

The respondents selected for this study were lecturers who have applied online teaching methods at NIPA School of Administration, comprising a total of 135 lecturers with nearly an equal proportion of female and male lecturers. Most of them are expert assistants with master's degrees. The respondent characteristics are shown in Table 2.

**Table 2:** Respondent characteristics.

Characteristic	Frequency	Percentage
**Gender**		
Man	66	48.9
Woman	69	51.1
**Academic Position**		
Expert Assistant	71	52.6
Lecturer	38	28.1
Head Lecturer	26	19.3
**Education**		
Master (S2)	92	68.1
Doctoral (S3)	43	31.9

### 3.3 Instruments development

Constructs in the preparation of the questionnaire were adapted from previous studies with slight modifications according to the research conditions. The instruments used in this study consisted of 18 main questions representing five variables (Table 3) consisting of ICT anxiety, Smartphone addiction, interruption, job efficacy and job performance adapted from a study by Prodanova & Kocarev (2021), one screening question, and four questions regarding the identity of the respondents. Each construct will be measured using a five-point Likert scale, namely Very Dissatisfied (1), Dissatisfied (2), Neutral (3), Satisfied (4), and Very Satisfied (5).

**Table 3:** Instruments.

No.	Indicator			Outer Loadings	Cronbach Alpha
1	IA	IA1	I feel **uncomfortable** with the electronic-based learning (e-learning) technology used/chosen by NIPA School of Administration.	.78	.81
2		IA2	In distance learning, I feel **worried** about pressing the wrong button, which can cause damage or loss of data stored in IT systems, both private and institutional.	.89	
3		IA3	I **doubt** using digital learning media, worried that uncorrectable error will occur.	.90	
4	SA	SA1	Using a smartphone is one of my main daily activities.	.96	.66
5		SA2	I feel concerned (insecure) supposing the smartphone is damaged or not working.	−.34 (deleted)	
6		SA3	I feel lost without my smartphone.	.72	
7	I	I1	I am easily distracted while teaching when it is conducted from home (distance learning).	.86	.87
8		I2	I feel that the working environment at home is not significantly supportive of the teaching process in distance learning.	.90	
9		I3	My concentration is hampered when teaching from home (distance learning).	.92	
10	JE	JE1	Work plans frequently change while teaching from home (distance learning).	.73	.79
11		JE2	I spend more time teaching with a distance learning method from home than face-to-face learning on campus.	.39 (deleted)	
12		JE3	I face many distractions in my daily work, including teaching from home (distance learning).	.87	
13		JE4	I dedicate more time when working from home, but complete less work.	.80	
14		JE5	My co-workers and I spend much time talking about our personal lives while working from home.	.74	
15	JP	JP1	Teaching from home (distance learning) helps me achieve my learning goals more efficiently than teaching on campus.	.86	.88
16		JP2	Teaching from home (distance learning) is more beneficial in improving **my performance** at NIPA School of Administration.	.83	
17		JP3	Teaching from home (distance learning) is more beneficial in improving **the performance of all lecturers** at NIPA School of Administration.	.91	
18		JP4	Teaching from home (distance learning) is more beneficial in providing ​**added value** ​for NIPA School of Administration (organizational profits).	.82	

To test the validity and reliability of all indicators, this study applied the Partial Least Square – Structural Equation Modeling (PLS-SEM) outer loadings with a minimum value of .7 (Hair et al., 2017b; Fitriati and Madu Siwi, 2022) and Cronbach's Alpha with a minimum value of .6 (Malhotra, 2020; Fitriati and Madu Siwi, 2022) respectively. All indicators obtain Cronbach's Alpha values of 0.7–0.8 (Table 3). By referring to convergent validates, an indicator is declared valid if the loading factor value is .5 (Hair et al., 2017a). The results of the validity test are presented in Table 3.

### 3.4 Questionnaire administration procedure

The questionnaire requires approximately 5–10 min to fill since it consists of 18 closed questions. The questionnaire link was shared to the coordinator and developed for all lecturers at NIPA School of Administration. Respondents can open the questionnaire on Google Forms via the link shared. The questionnaire consists of 18 questions as follows: three questions for ICT anxiety, three questions for smartphone addiction, three questions for interruption, five questions for job efficacy, and four questions for job performance. The 18 questions are validated through the PLS-SEM outer loadings test, resulting in the deletion of SA2 and JE2 items. Data collection was conducted from September to October 2021.

### 3.5 Measures and statistical analyses

The data collected will later go through a validity test using outer loadings with a minimum value of .7 (Hair et al., 2017b) as well as a reliability test using Cronbach's Alpha with a minimum value of .6 (Malhotra, 2020). Considering the small number of sample used in this study (Hair et al., 2017b), the statistical tests applied PLS-SEM using SMARTPLS3 software. PLS-SEM has unlimited algorithms for reflective and formative latent constructs and can estimate very complex path and study models. A very complex study model consists of many latent and manifest variables without experiencing problems when estimating data. Furthermore, PLS-SEM can be used when data distribution is not spread across the mean values. Hair et al., 2017b further explain that PLS-SEM is divided into two types: the outer and inner models.

The statements of the respondents in the questionnaire were summarized and described in the form of mean and standard deviation values. The indicators of the study obtained a mean of 3.11, implying the neutral opinion of the respondents regarding the influence of ICT anxiety and smartphone addiction on job performance. Meanwhile, the average value of standard deviation of all indicators is 1.07, concluding the mean score in the range of 3.11 ± 1.07. The descriptive statistic for each research indicator is presented in Table 4.

**Table 4:** Descriptive statistics of research indicators.

No	Indicators	N	Mean	Standard Deviation
1	**I1**	135	3.47	1.26
2	**I2**	135	3.71	1.15
3	**I3**	135	3.87	1.12
4	**IA 1**	135	3.96	1.13
5	**IA 2**	135	3.90	1.13
6	**IA 3**	135	4.14	.96
7	**JE1**	135	2.31	1.13
8	**JE3**	135	2.25	1.12
9	**JE4**	135	2.22	1.08
10	**JE5**	135	1.63	.86
11	**JP1**	135	3.57	1.06
12	**JP2**	135	3.53	1.12
13	**JP3**	135	3.46	1.05
14	**JP4**	135	3.69	.99
15	**SA1**	135	1.77	.91
16	**SA3**	135	2.36	1.11
Mean			3.11	1.07

## 4 Results

### 4.1 Assessment of the measurement or outer model

**Figure 2:**
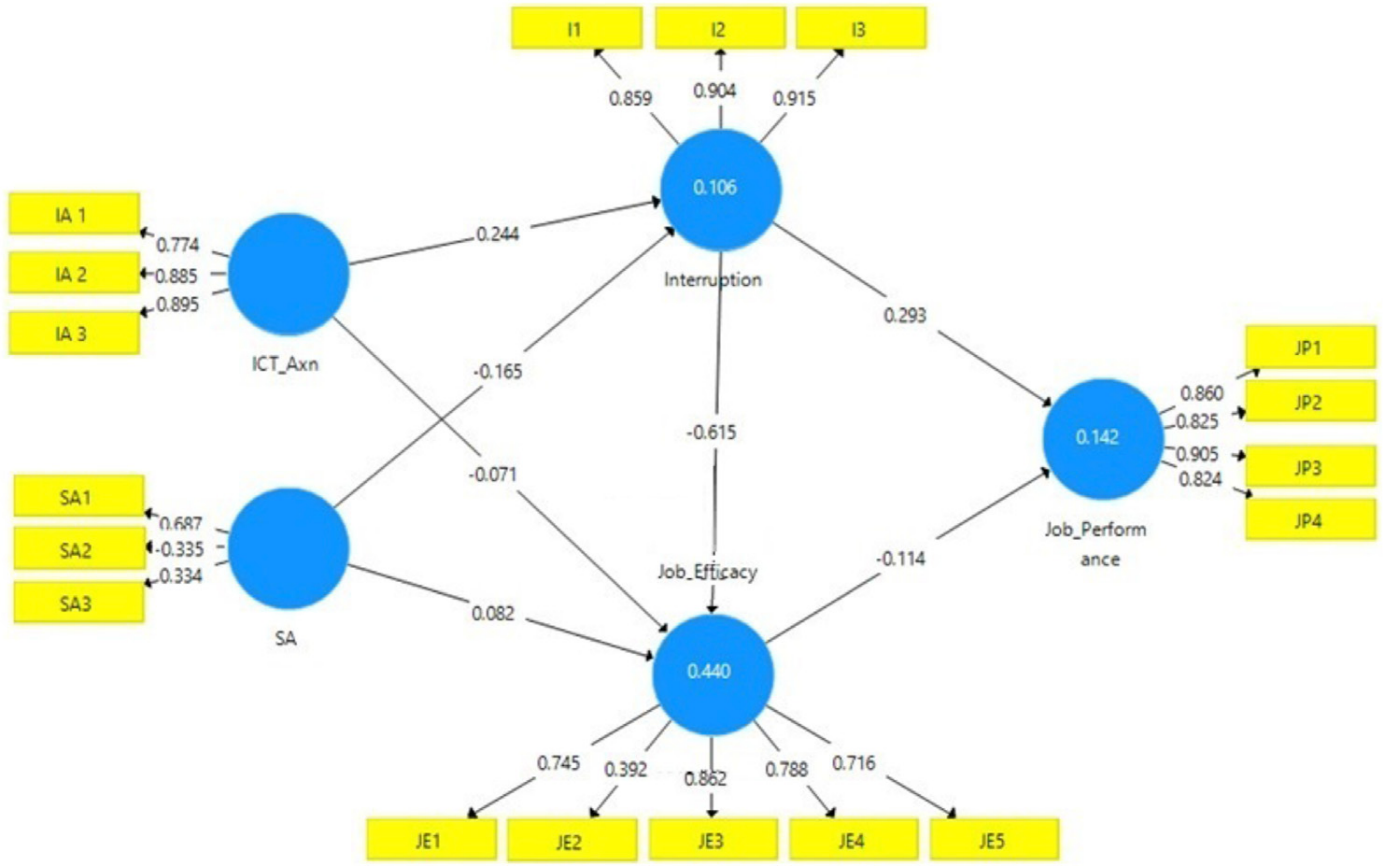
Full model confirmatory factor analysis (CFA)

The assessment of the outer model can be grouped into two, namely validity and reliability tests, each of which will lead to several more tests. All values of the outer model tests are shown in Table 5 and Figure 2. The first validity test is convergent validity, which proves that the respondents can understand all statements on the latent variables. Convergent validity refers to outer loadings of more than .7 (Hair et al., 2017b) and AVE.

**Table 5:** Internal consistency measures for measurement model.

Variable	Indicator	Statement	(λ)	CR	α	AVE
**ICT Anxiety (IA)**	IA1	I feel **uncomfortable** with the electronic-based learning (e-learning) technology used/chosen by NIPA School of Administration.	.78	.89	.81	.73
	IA2	In distance learning, I feel **worried** about pressing the wrong button, which can cause damage or loss of data stored in IT systems, both private and institutional.	.88			
	IA3	I **doubt** using digital learning media, worried that uncorrectable error will occur.	.89			
**Smartphone Addiction (SA)**	SA1	Using a smartphone is one of my main daily activities.	.96	.83	.66	.72
	SA3	I feel lost without my smartphone.	.72			
**Interruption (I)**	I1	I am easily distracted while teaching when it is conducted from home (distance learning).	.78	.99	.87	.80
	I2	I feel that the working environment at home is not significantly supportive of the teaching process in distance learning.	.88			
	I3	My concentration is hampered when teaching from home (distance learning).	.89			
**Job Efficacy (JE)**	JE1	Work plans frequently change while teaching from home (distance learning).	.73	.86	.79	.62
	JE3	I face many distractions in my daily work, including teaching from home (distance learning).	.87			
	JE4	I dedicate more time when working from home, but complete less work.	.80			
	JE5	My co-workers and I spend much time talking about our personal lives while working from home.	.74			
**Job Performance (JP)**	JP1	Teaching from home (distance learning) helps me achieve my learning goals more efficiently than teaching on campus.	.86	.92	.88	.73
	JP2	Teaching from home (distance learning) is more beneficial in improving **my performance** at NIPA School of Administration.	.83			
	JP3	Teaching from home (distance learning) is more beneficial in improving **the performance of all lecturers** at NIPA School of Administration.	.90			
	JP4	Teaching from home (distance learning) is more beneficial in providing ​**added value** ​for NIPA School of Administration (organizational profits).	.82

In the initial calculation, three indicators obtain outer loading values of less than 0.7, namely SA2 (−.34), SA3 (−.33) and JE2 (.39). Thus, SA2 indicator is removed. In the next measurement, SA3 obtains a value of .72 while JE2 is constant at .39. Thus, these indicators are also deleted. The negative outer loading value of SA2 shows a negative correlation of SA2 with smartphone addiction. Furthermore, removing SA2 from the model can increase the outer loadings value of SA1 and SA3. It is similar to the exclusion of JE2 that increases the reliability value of JE3, JE4, and JE5.

The AVE values obtained, which are more than 0.5, show that all variables fall into the valid category. The AVE value of each variable is as follows: .73 for ICT anxiety, .72 for smartphone addiction, .80 for interruption, .62 for job efficacy, and .73 for job performance. Thus, the lowest AVE value is obtained by job efficacy while the highest AVE value is obtained by interruption.

The second validity test is discriminant validity to prove that the respondents do not confuse the statement on the latent variable with questions on the other latent variables, particularly in terms of meaning. Discriminant validity is met supposing the HTMT value is less than .90. Referring to Table 6, all HTMT values are less than .90, thus discriminant validity through HTMT is said to be valid (Henseler et al., 2015).

**Table 6:** Results of HTMT measurement.

	ICT Anxiety	Interruption	Job Efficacy	Job Performance	Smartphone Addiction
ICT Anxiety					
**Interruption**	.34				
**Job Efficacy**	.32	.77			
**Job Performance**	.13	.42	.37		
**Smartphone Addiction**	.32	.12	.17	.42	

The next assessment of the outer model is internal consistency reliability using two values, namely composite reliability and Cronbach's alpha. Most variables (ICT anxiety, interruption, job efficacy, and job performance) obtain a Cronbach's alpha value ranging from .70 to .90 while smartphone addiction obtains a Cronbach's alpha value ranging from .60 to .70. Thus, these variables are acceptable. In addition to Cronbach's alpha, this study also used a composite reliability value, in which all variables obtain a value of more than .70. In addition to measuring validity and reliability, the assessment of the outer model also pays attention to the multicollinearity test using the VIF value. Based on Tables 7 and 8, it is obvious that the VIF value is less than five, indicating collinearity between constructs.

**Table 7:** Results of outer VIF measurement.

Indicators	VIF
**I1**	1.99
**I2**	2.64
**I3**	2.70
**IA 1**	1.49
**IA 2**	2.12
**IA 3**	2.25
**JE1**	1.54
**JE3**	2.11
**JE4**	1.57
**JE5**	1.47
**JP1**	2.15
**JP2**	2.06
**JP3**	2.89
**JP4**	2.00
**SA1**	1.31
**SA3**	1.31

**Table 8:** Inner VIF values.

	ICT Anxiety	Interruption	Job Efficacy	Job Performance	Smartphone Addiction
**ICT Anxiety**		1.07	1.15		
**Interruption**			1.09	1.73	
**Job Efficacy**				1.73	
**Job Performance**					
**Smartphone Addiction**		1.07	1.07		

### 4.2 Assessment of the structural or inner model

The inner model analysis begins with the R-Square (*R2*) test, which aims to determine whether the endogenous latent variable has predictive power to the model or not (Handayati et al., 2020) or whether the *R2* value indicates accuracy predictions or not (Hair et al., 2013). The rule of thumb for an acceptable R2 value is .67, .33, and .19, respectively explained as substantial, moderate, and weak (Chin, 2010).

As shown in Table 9, ICT anxiety and smartphone addiction influence interruption by .08 or 8.0%. Then ICT anxiety, smartphone addiction, and interruption influence job efficacy by .43 or 43%. Lastly, interruption and job efficacy influence job performance by .15 or 15%. The second test of the inner model is confidence intervals, whose results are shown in Table 10. The value of confidence intervals is 97.5%, thus the mean range of the population will fall between −.483 to .476.

**Table 9:** Variance explained by the model.

	R-Square	R Square Adjusted
**Interruption**	.08	.07
**Job Efficacy**	.43	.42
**Job Performance**	.15	.13

**Table 10:** Values of predictive relevance from the model.

	SSO	SSE	Q^2^ (=1 − SSE/SSO)
**ICT anxiety**	405.000	405.000	
**Interruption**	405.000	382.137	.06
**Job Efficacy**	540.000	408.350	.24
**Job Performance**	540.000	486.428	.10
**Smartphone Addiction**	270.000	270.000	

The next test is the effect size (*f2*) with the rule of thumb refers to Cohen (2013) and Hair et al. (2014), namely the values of .02, .15, and .35 to show small, medium, and large effect sizes, respectively. The effect of a specific exogenous construct on the endogenous construct can be assessed by evaluating the f2 effect sizes. Eliminating the effect size of each exogenous variable on the explanatory power of the model reveals that eliminating the exogenous variables (ICT anxiety and smartphone addiction) that explain interruption has a small effect size (.08 and .00, respectively). Eliminating interruption and job efficacy that explain job performance has a small effect size (.05 and 0.01, respectively) while removing ICT anxiety and smartphone addiction that explain job efficacy has a small effect size (.00 and .01, respectively). Furthermore, removing interruption that explains job efficacy has a high effect size (.64).

Furthermore, predictive relevance (*Q2*) using blindfolding is carried out with the obtained value of .06 for interruption, .24 for job efficacy, and .10 for job performance (Table 10). It is evident that the value of *Q2* > 0, indicating that the model has predictive relevance.

**Figure 3:**
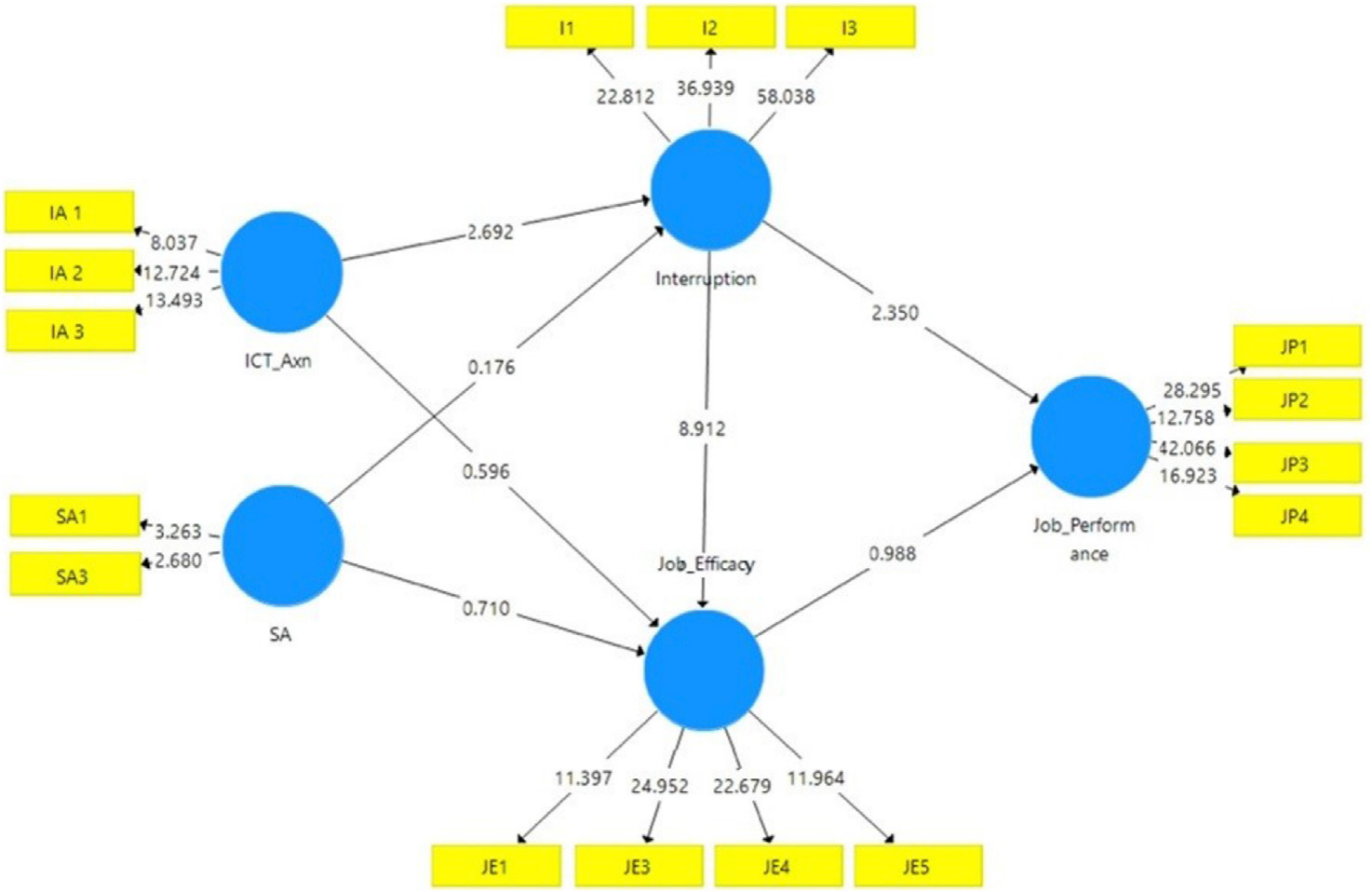
Bootstrap image for path analysis.

The last analysis in the inner model is the path coefficient with the following results (Figure 3 and Table 11): (a) the relationship between ICT anxiety and interruption is positive (.28); (b) the relationship between smartphone addiction and interruption is negative (−.02); (c) the relationship between ICT anxiety and job efficacy is negative (−.05); (d) the relationship between smartphone addiction and job efficacy is positive (.06); (e) the relationship between interruption and job efficacy is negative (.63); (f) the relationship between interruption and job performance is positive (.28); and (g) the relationship between job efficacy and job performance is negative (−.14).

**Table 11:** Results of path analysis.

Hypotheses	Β	P Values	f2 values	Confidence interval		Decision
				LL	UL	
**ICT anxiety**–**> Interruption**	.28	.01	.08	.07	.48	**Accepted**
**ICT anxiety**–**> Job Efficacy**	−.05	.55	.00	−.24	.14	Rejected
**Interruption**–**> Job Efficacy**	−.63	.00	.64	−.75	−.48	**Accepted**
**Interruption**–**> Job Performance**	.28	.02	.05	.04	.54	**Accepted**
**Job Efficacy**–**> Job Performance**	−.14	.32	.01	−.40	.16	Rejected
**Smartphone Addiction**–**> Interruption**	−.02	.86	.00	−.25	.20	Rejected
**Smartphone Addiction**–**> Job Efficacy**	.06	.48	.01	−.10	.21	Rejected

The next stage examines the *p-values*, from which it is concluded that three of seven hypotheses are accepted:

1. The first hypothesis, stating that ICT anxiety has positive influences on interruption, is **accepted** (*p-value*: .01).
2. The second hypothesis, stating that ICT anxiety has negative influences on job efficacy, is rejected (*p-value*: .55).
3. The third hypothesis, stating that smartphone addiction has positive influences on interruption, is rejected (*p-value*: .86).
4. The fourth hypothesis, stating that smartphone addiction has negative influences on job efficacy, is rejected (*p-value*: .48).
5. The fifth hypothesis, stating that interruption has negative influences on job efficacy is **accepted** (*p-value*: .00).
6. The sixth hypothesis, stating that interruption has negative influences on job performance, is **accepted** (*p-value*: .02).
7. The seventh hypothesis, stating that job efficacy has positive influences on job performance, is rejected (*p-value*: .32).

This study also conducted bootstrapping, a non-parametric approach to test the accuracy/precision of PLS-SEM testing (Henseler et al., 2015). Bootstrapping allows testing the statistical significance of various PLS-SEM results such as path coefficients, Cronbach's alpha, *HTMT*, and *R*^*2*^. Bootstrapping is carried out supposing the data already meet the criteria in the outer model.

## 5 Discussion

This section discusses the findings obtained by relating them to the theory that has been built. Despite the current digital era where technology is commonplace, the use of technology in the work environment is one of the challenging issues as technology can cause negative impacts. However, on the other hand, technology has a highly positive impact on work activities. These negative impacts include interference from the internal environment and even from technological tools. These two sides, namely the negative and positive impacts of technology, challenge workers to maintain and even improve their performance (Lowe-Calverley and Pontes, 2020). Following the explanation in the Introduction section, this study aims to analyze the impact of ICT anxiety and smartphone addiction on job performance of all lecturers at NIPA School of Administration with intervening variables, namely interruption and work efficacy. Between humans and technology, interactions have been created, one of which is in the world of work. Therefore, this study aims to observe the effect of technology described in ICT anxiety and smartphone addiction on work performance.

In this study, job demands are represented by ICT anxiety as the lecturers are demanded to understand technology in the era of distance learning, while resources are represented by smartphone addiction. Increased ICT anxiety can increase work disorders since the lack of individual self-confidence and increased fear and discomfort when using technology can hinder work activities (Celik and Yesilyurt, 2013; Meuter et al., 2003). The results of hypothesis testing using PLS-SEM show that there is a positive relationship between ICT anxiety and interruption while interruption has negative influences on job efficacy and job performance.

The results obtained from the questionnaire in terms of ICT anxiety show that only a small group of lecturers at NIPA School of Administration feel uncomfortable, worried, and doubtful when using technology in distance learning activities because they are accustomed to using technology in work activities. Therefore, low ICT anxiety of lecturers at NIPA School of Administration will also decrease work disturbances caused by the use of technology during distance learning. Technology is not an obstacle to the work of lecturers at NIPA School of Administration but can be a means of more efficient work activities (Mac Callum et al., 2014). Based on the answers of the respondents in the explanation section, it can be concluded that low ICT anxiety of the lecturers is also influenced by technology socialization and the readiness of the IT team to assist in distance learning activities.

Lecturers at NIPA School of Administration frequently experience interruption while teaching from home, for instance, distracted by family or bad internet connection. In this regard, interruption can decrease job efficacy and job performance. Technology use without preparation and evaluation, lack of digital skills, and the emergence of other technology use disorders will cause high interruption in the implementation of online learning systems (Oliveira et al., 2021).

However, despite having several obstacles, the lecturers at NIPA School of Administration consider that online teaching activities have become a habit that provides convenience and benefits, particularly during the pandemic. Preparation for the use of technology in learning activities carried out by permanent lecturers at NIPA School of Administration forms a process of adaptation and resilience to the use of technology to a large extent to minimize interruption (Latchem, 2014; Stein et al., 2007). With good ICT anxiety conditions, permanent lecturers at NIPA School of Administration tend to explore other technologies to balance and even increase distance learning activities (Balta-Ozkan et al., 2013; Venkatesh et al., 2012).

The significance of the relationship between interruption and a decrease in job efficacy and job performance is in line with the findings of Prodanova and Kocarev (2021), which explain that work disorders influence the efficacy and performance of work activities of an individual, hence the necessity for the individual to be able to control possible disturbances that can occur.

Another finding shows that smartphone addiction has no negative relationship with interruption or job efficacy. The use of smartphones by permanent lecturers at NIPA School of Administration is described as a form of work activity because it helps establish communication and uncomplicated data storage media and helps carry out work activities anywhere and anytime. This finding is in line with the opinion of Duke and Montag (2017) that using smartphones can increase work activities and provide motivation, allowing workers to carry out work activities more effectively and efficiently, despite the relatively small effect. The high use of smartphones to help work activities can produce much better output.

In terms of effectiveness and efficiency, the job performance of lecturers at the NIPA School of Administration tends to increase during distance learning, albeit not significant when compared to direct teaching. According to Murphy (Dillon and Pellegrino, 1989), the factors that influence job performance are the working behavior towards tasks. In this regard, distance learning activities carried out by lecturers are not much different from direct and interpersonal teaching activities. In relation to behavior, communication barriers can occur between lecturers and students due to increasingly limited interactions and the inability of lecturers to pay attention to students. Several disturbances that can hinder work activities of the lecturers also tend to occur from external factors, such as inadequate internet networks and home environmental factors. Organizations can benefit through the unique involvement and collaboration of each party for organizational governance. The benefits of increased company performance can be obtained through a collaborative process (Budiarso et al., 2021).

The permanent lecturers at NIPA School of Administration consider that the use of technology in the distance teaching system has been effective and supporting teaching activities. In this regard, it is evident that the role of the campus in terms of socialization is one of the keys to creating good relations between permanent lecturers at NIPA School of Administration and the application of technology.

## 6 Conclusion

The COVID-19 pandemic has a great impact on the implementation of the distance learning system for NIPA School of Administration. Although lecturers and students have started to get used to the online learning system, the communication and interaction between lecturers and students are not adequate. This study also shows a significant impact of ICT anxiety on interruption and interruption on job efficacy and job performance. However, the impact of other variables such as smartphone addiction on interruption and job efficacy, ICT anxiety on job efficacy, and job efficacy on job performance is not significant.

Therefore, this study recommends the facilitation of knowledge sharing related to ICT competence or literacy. For instance, personal sharing by young lecturers with senior lecturers in addition to mutual training and not generalizing the competence (digital literacy) of each lecturer. Second, improving the security guarantees of the intellectual rights of the lecturers in relation to the choice of technology provided by campus. Third, integrating the demands of ICT needs with administrative-technical procedures, particularly in financing/budgeting aspect. For instance, students demand the availability of video-based learning that they can repeatedly access, yet it is constrained by financial administration procedures.

This study has several limitations. First, the sample used is the lecturers at the NIPA School of Administration, rendering the sample unable to be replicated by lecturers with various levels of office in other areas or other universities or even by other professions. Thus, future studies can use a representative sample. Second, the sample size is limited due to the small size of the total population of this study. A large data size can show more statistical power*.* Third, the data were obtained only from questionnaire, thus future studies can include interviews to strengthen the analysis. Fourth, this study renders the impact of using technology on job performance only during the period of the study. Meanwhile, there have been numerous changes during the pandemic, which may lead to exciting findings. Future studies can apply a longitudinal study instead. Finally, several results of this study need to be further studied.

## Declarations

### Author contribution statement

Adi Suryanto, Rachma Fitriati, Sela Inike Natalia, Andina Oktariani, Munawaroh, Nurliah Nurdin, Young-hoon AHN: Conceived and designed the experiments; Performed the experiments; Analyzed and interpreted the data; Contributed reagents, materials, analysis tools or data; Wrote the paper.

### Funding statement

This work was supported by Program Hibah Publikasi Terindeks Internasional (PUTI) Q1 Tahun Anggaran 2022-2023 Nomor: NKB-386/UN2.RST/HKP.05.00/2022.

### Data availability statement

Data included in article/supp. material/referenced in article.

### Declaration of interest’s statement

The authors declare no conflict of interest.

### Additional information

No additional information is available for this paper.
